# 1,25-Dihydroxyvitamin D Deficiency Accelerates Aging-related Osteoarthritis via Downregulation of Sirt1 in Mice

**DOI:** 10.7150/ijbs.78785

**Published:** 2023-01-01

**Authors:** Jie Chen, Jiao Zhang, Jie Li, Ran Qin, Na Lu, David Goltzman, Dengshun Miao, Renlei Yang

**Affiliations:** 1 Department of Plastic Surgery, Affiliated Friendship Plastic Surgery Hospital of Nanjing Medical University, Nanjing Medical University, Nanjing, China.; 2 The Research Center for Bone and Stem Cells, Department of Anatomy, Histology and Embryology, Nanjing Medical University, Nanjing, China.; 3 Center for Experimental Medicine, The Third Xiangya Hospital, Central South University, Changsha, China.; 4 Calcium Research Laboratory, McGill University Health Centre and Department of Medicine, McGill University, Montreal, Quebec H4A 3J1, Canada.

**Keywords:** Vitamin D deficiency, vitamin D supplementation, vitamin D receptor, osteoarthritis, Sirt1

## Abstract

Emerging observational data suggest that vitamin D deficiency is associated with the onset and progression of knee osteoarthritis (OA). However, the relationship between vitamin D level and OA and the role of vitamin D supplementation in the prevention of knee OA are controversial. To address these issues, we analyzed the articular cartilage phenotype of 6- and 12-month-old wild-type and 1α(OH)ase^-/-^ mice and found that 1,25(OH)_2_D deficiency accelerated the development of age-related spontaneous knee OA, including cartilage surface destruction, cartilage erosion, proteoglycan loss and cytopenia, increased OARSI score, collagen X and Mmp13 positive chondrocytes, and increased chondrocyte senescence with senescence-associated secretory phenotype (SASP). 1,25(OH)_2_D_3_ supplementation rescued all knee OA phenotypes of 1α(OH)ase^-/-^ mice *in vivo*, and 1,25(OH)_2_D_3_ rescued IL-1β-induced chondrocyte OA phenotypes *in vitro*, including decreased chondrocyte proliferation and cartilage matrix protein synthesis, and increased oxidative stress and cell senescence. We also demonstrated that VDR was expressed in mouse articular chondrocytes, and that VDR knockout mice exhibited knee OA phenotypes. Furthermore, we demonstrated that the down-regulation of Sirt1 in articular chondrocytes of 1α(OH)ase^-/-^ mice was corrected by supplementing 1,25(OH)_2_D_3_ or overexpression of Sirt1 in mesenchymal stem cells (MSCs) and 1,25(OH)_2_D_3_ up-regulated Sirt1 through VDR mediated transcription. Finally, we demonstrated that overexpression of Sirt1 in MSCs rescued knee OA phenotypes in 1α(OH)ase^-/-^ mice. Thus, we conclude that 1,25(OH)_2_D_3,_ via VDR-mediated gene transcription, plays a key role in preventing the onset of aging-related knee OA in mouse models by up-regulating Sirt1, an aging-related gene that promotes articular chondrocyte proliferation and extracellular matrix protein synthesis, and inhibits senescence and SASP.

## Introduction

Osteoarthritis (OA) characterized by pain, progressive loss of articular cartilage and structural changes is extremely common in aged individuals 1. Vitamin D deficiency has also been a common health problem. Over a billion people worldwide are vitamin D deficient or insufficient 2. Vitamin D deficiency is closely associated with common chronic diseases such as metabolic bone disorders, diabetes and cardiovascular diseases 3. Emerging observational data suggest that vitamin D insufficiency is associated with the onset and progression of knee OA 4-6. However, studies examining the prevention of OA after vitamin D supplementation have been inconclusive 7. A pilot randomized controlled trial in India revealed that 12-months of vitamin D treatment had small but statistically significant clinical benefits on pain and function in patients with knee OA compared with placebo 8. In contrast, a subsequent randomized controlled trial in the United States showed that vitamin D supplementation over 24 months did not reduce knee pain or cartilage volume loss 9. Subsequently, data from randomized controlled trials in Australia 10 and the UK 11 did not find a significant clinical benefit of vitamin D supplementation for knee OA. Well-designed animal studies are required to investigate the role of vitamin D in the initiation and progression of knee OA; however, adequate cell and animal models are lacking.

Vitamin D, mainly synthesized in skin tissue, is firstly metabolized by liver 25-hydroxylase, to 25-hydroxyvitamin D [25(OH)D], which is then converted to 1,25(OH)_2_D by the renal or extrarenal 1α-hydroxylase [1α(OH)ase]. 1,25(OH)_2_D then exerts its principal actions by binding to the vitamin D receptor (VDR). Although 1,25(OH)_2_D deficient [1α(OH)ase^-/-^] 12 and VDR deficient (VDR^-/-^) 13 mouse models have been generated over 2 decades and have provided considerable insight into the regulation of mineral and skeletal physiology by 1,25(OH)_2_D 14, 15, whether OA phenotypes occur in long bone joints of 1α(OH)ase^-/-^ and VDR^-/-^ mice has not been reported. We have previously reported that 1,25(OH)_2_D deficiency may play a role in the pathogenesis of temporomandibular joint OA 16. However, it is unknown whether 1,25(OH)_2_D deficiency can induce the occurrence of knee OA via VDR and what the key downstream targets of 1,25(OH)_2_D might be in preventing the occurrence of knee OA.

Sirt1 is a mammalian homologue of Sir2, which is a histone deacetylase that regulates gene expression and protein function by deacetylating lysine residues in histone and non-histone proteins, including p65 and forkhead box protein O. Sirt1 exerts anti-aging and other protective effects through regulation of mitochondrial biogenesis, oxidative stress, and inflammation-pathways that are known to drive OA 17. Sirt1 has a chondroprotective role by suppressing interleukin-1β (IL-1β)- and tumor necrosis factor α-induced expression of cartilage-degrading enzymes. In mice, Sirt1 haploinsufficiency 18 or Sirt1-inactivating mutations 19 results in delayed growth and increased spontaneous OA. Chondrocyte-specific deletion of Sirt1 resulted in increased severity of OA with aging and following joint injury 20. These findings in mice are also consistent with the reduction of Sirt1 expression in human OA cartilage. However, it is unclear whether Sirt1 is a key downstream target for 1,25(OH)_2_D in preventing the occurrence of knee OA.

Previous studies have shown that cells isolated from the superficial zone of mouse and human articular cartilage express mesenchymal stem cell (MSC) markers and exhibit MSC characteristics, and MSCs have been a therapeutic option for the prevention of osteoarthritis 21-23. Indeed, accumulation of senescent MSCs in joints contributes to the development of osteoarthritis, and recent studies have reported that the transplantation of MSCs or the rejuvenation of endogenous senescent MSCs could be a therapeutic option for osteoarthritis 21-23. Prx1-expressing periosteal cells can differentiate into chondrocytes and osteoblasts *in vitro* and Prx1 has been used to study chondrogenesis and/or skeletal limb formation. Therefore, periosteal Prx1-expressing cells are most likely chondro-osteoprogenitor cells 24. Recently, we generated a transgenic mouse model by overexpressing Sirt1 in MSCs using Prx1 as a promoter (Sirt1^Tg^) and found that overexpression of Sirt1 in MSCs protected against bone loss induced by Bmi1 deficiency in long bone and mandibles 25, 26 and improved the mandibular bone loss induced by 1,25(OH)_2_D deficiency 27 without altering mineral levels.

In our current study, to investigate the mechanism of 1,25(OH)_2_D deficiency in the pathogenesis of knee OA, we firstly compared the articular cartilage phenotype of 6- and 12-month-old wild-type and 1α(OH)ase^-/-^ mice fed a high calcium/phosphate (rescue) diet to assess whether 1,25(OH)_2_D deficiency induced age-related spontaneous knee OA. We then treated 1α(OH)ase^-/-^ mice or IL-1β-treated human articular chondrocytes with exogenous 1,25(OH)_2_D_3_ to assess whether treatment with 1,25(OH)_2_D_3_ rescued the knee OA phenotype caused by 1,25(OH)_2_D deficiency, and also the IL-1β-induced reduction of chondrocyte proliferation and the increases of oxidative stress and cellular senescence. We also compared the articular chondrocyte and cartilage phenotypes of 6-month-old wild-type and VDR^-/-^ mice to assess whether VDR deficiency induced chondrocyte senescence and a knee OA phenotype. The mechanisms of 1,25(OH)_2_D_3_ in regulating Sirt1 via VDR were examined in chondrocytes using molecular techniques. Finally, we generated 1α(OH)ase^-/-^ mice with Sirt1 overexpression in MSCs, and compared their knee phenotypes with 1α(OH)ase^-/-^ mice on the rescue diet and wild-type littermates to assess whether overexpression of Sirt1 in MSCs could prevent 1,25(OH)_2_D deficiency-induced development of knee OA.

## Materials and Methods

### Animals and treatment

The generation and characterization of 1α(OH)ase^-/-^ mice and vitamin D receptor knockout mice (VDR^-/-^) were previously described 28. After weaning, male 1α(OH)ase^-/-^ mice and wild-type (WT) littermates were given a rescue diet (RD) containing 2.0% calcium, 1.25% phosphorus and 20% lactose and mice aged 6 and 12 months were sacrificed for joint phenotype analysis. WT and 1α(OH)ase^-/-^ mice on a normal diet were subcutaneously injected, respectively, with vehicle and 1,25(OH)_2_D_3_ (1 μg/kg B.W.) three times per week, from the time of weaning until analysis at the age of 12 months. WT and VDR^-/-^ littermates were given the rescue diet after weaning and sacrificed at the age of 6 months for joint phenotype analysis. The transgenic mouse model of Sirt1 overexpression driven by Prx1 gene, which represents the mesenchymal lineage (Sirt1^Tg^) was generated as previously described 29. Meanwhile, 1α(OH)ase^+/-^ mice and Sirt1^Tg^ mice were mated to produce offspring heterozygous at both loci (1α(OH)ase^+/-^Sirt1^Tg^), which were then mated to generate 1α(OH)ase^-/-^Sirt1^Tg^ mice. After weaning, WT, Sirt1^Tg^, 1α(OH)ase^-/-^ and 1α(OH)ase^-/-^Sirt1^Tg^ mice were given the rescue diet and used for phenotype analysis at the age of 12 months. All animal experiments were performed in compliance with the guidelines approved by the Institutional Animal Care and Use Committee of Nanjing Medical University.

### Measurements of serum calcium, phosphorus, PTH, 25(OH)D and 1,25(OH)_2_D

Serum calcium and phosphorus levels were analyzed by an autoanalyzer (Beckman Synchron 67; Beckman Instruments). Serum intact PTH was measured using an ELISA kit (Immutopics, Inc., San Clemente, CA). The serum levels of 25(OH)D_3_ (Nanjing Jiancheng Bioengineering Institute) and 1,25(OH)_2_D (Nanjing Jiancheng Bioengineering Institute) were measured as previously described 30.

### Histological evaluation of articular cartilage

At the time of euthanasia, mouse knee joints were dissected free of soft tissue and fixed in PLP fixative buffer for 48 hours at 4°C, decalcified with 10% EDTA and embedded in paraffin, after which 5-μm sections were stained for safranin O/fast green. The histological OA severity was evaluated using the Osteoarthritis Research Society International (OARSI) scoring system (grade 0-6). For each joint, OA grade was examined by a single observer under blinded conditions.

### Microtomography (μ-CT)

Knee joints of indicated groups were collected and examined using μCT and subchondral bone volume were examined as previously described 31.

### Articular chondrocyte cultures and treatment

Human articular chondrocytes were purchased from Procell (Wuhan, China). Primary mouse chondrocytes isolated from articular cartilage of newborn mice were cultured as described previously. Briefly, cartilage tissue was cut into 1 mm^3^ pieces, washed three times with PBS and digested with 0.2% type II collagenase (Worthington Biochemical, 4176) in high-glucose DMEM-F12 (Gibco) overnight at 37 °C. After digestion, the filtrate was passed through a 70-µm strainer, and cells were washed three times with PBS, and seeded in DMEM-F12 complete medium containing 100 U/ml penicillin, 100 µg/ml streptomycin and 10% FBS (Gibco) at 37 °C with 5% CO_2_. For 3D cultures of human and mouse chondrocytes, 400,000 cells were seeded in 96-well MicroWell round-bottom plates (Thermo Fisher Scientific). Pellets were obtained after centrifugation at 150g for 10 min. The chondrocyte pellets were maintained at 37 °C with 5% CO_2_ in 200µl of chondrocyte growth medium consisting of high-glucose DMEM-F12, 1% Pen/Strep and 10% FBS. Recombinant Human Interleukin-1β (IL-1β) (200-01B, PeproTech), at 10ng/ml, was added as an inflammatory stimulus, and 100nM 1,25(OH)_2_D_3_ and control vehicle were added into indicated wells. Pellets were harvested after 21 days for evaluation. Medium was changed every other day until the end of the experiment. SA-β-gal staining, crystal violet staining, EdU assay and dihydroethidium (DHE) staining were performed as previously described 32. Ex527, a selective Sirt1 inhibitor was purchased from MedChemExpress (MCE).

### Immunofluorescence and immunohistochemistry staining

For immunofluorescence staining, cultured cells and sections were incubated with rabbit anti- VDR (Abcam, ab3508) at 4 °C overnight followed by using Alexa Fluor Plus 594 goat anti-rabbit IgG (H+L) secondary antibody (Invitrogen, A32740, 1:500 dilution) to detect immunoreactivity. For Immunohistochemistry staining, dewaxed and rehydrated paraffin-embedded sections were incubated with 6% hydrogen peroxide to block endogenous peroxidase activity and then washed in PBS (pH 7.6). The slides were then incubated with the primary antibodies, including rabbit anti-VDR (Abcam, ab3508), rabbit anti-β-gal (Abcam, ab616), rabbit anti-Collagen II (Abcam, ab34712), mouse anti-Collagen X (Abcam, ab49945), rabbit anti-Mmp13 (Proteintech, 18165-1-AP) and mouse anti-p16 (Santa Cruz, sc-1661) overnight at 4 °C. After rinsing with PBS for 15 min, sections were incubated with secondary antibody (biotinylated goat anti-rabbit IgG and goat anti-mouse IgG, Sigma). Sections were then washed and incubated with the Vectastain Elite ABC reagent (Vector Laboratories) for 30 minutes. Staining was done using 3,3-diaminobenzidine (2.5 mg/ml) followed by counterstaining with Mayer's hematoxylin.

### ChIP-qPCR

Chromatin immunoprecipitation (ChIP) was performed using the ChIP kit (Millipore, USA) as previously described 33. Briefly, human chondrocytes were treated with vehicle or 10^-7^ M 1,25(OH)_2_D_3_ for 3 hours, and then cell samples were subjected to immunoprecipitation using either a control IgG or rabbit anti-VDR antibody (Abcam, ab3508). The co-precipitated chromatin was determined by qPCR for the presence of human Sirt1 promoter sequence using Sirt1 sense 5′-TTAGAGTGGCTTACAGGC-3′ and antisense 5′-ACATCTTCTGGCTTCCTT-3′ primers.

### Luciferase reporter assay

To generate Sirt1 promoter-activated luciferase reporter, -1442 to +97 bp of Sirt1 promoter and the deletion mutant were directly synthesized and cloned into pGL3-basic. Luciferase reporter assay was performed as previously described 33. Briefly, human chondrocytes (passage 4) in 12-well plates were transfected with Sirt1-promoter or Sirt1-promoter mutant luciferase reporter plasmid. Twelve hours later, the medium was changed and the indicated concentration of vehicle or 1,25(OH)_2_D_3_ were added. After another 36 hours, luciferase activity was measured using the Dual-Luciferase assay kit (Promega). pRL-TK was co-transfected to normalize transfection efficiency.

### Western blots

Cell or tissue lysates were extracted for loading into 10% SDS-PAGE gels and immunoblotting was performed as previously described 34. Primary antibodies, including rabbit anti-Cyp27b1/1α(OH)ase (Abcam, ab206655), rabbit anti-VDR (Abcam, ab3508), rabbit anti-Sirt1 (Millipore, 07-131), rabbit anti-p16 (Proteintech, 10883-1-AP) and rabbit anti-β-actin (Cell Signaling Technology, 8457S) were used for immunoblotting. Immunoreactive bands were visualized with ECL chemiluminescence (Bio-Rad) and analyzed by Image J.

### RNA isolation and real-time RT-PCR

RNA isolation from cells and joint tissue, cDNA synthesis and quantitative real-time PCR (qRT-PCR) were performed as previously described 32. The PCR primer sequences used in this study are shown in the Supplementary Table 1.

### Statistical analysis

Measured data were described as mean ± SD. The statistical analyses were performed using GraphPad Prism (Version 8.0). The number of animals used in the experiments *in vivo* was shown in indicated figure legends. Two-tailed Student's t-test was used to compare differences between groups. For multiple comparisons, one-way ANOVA analysis of variance followed by Tukey's post-hoc test was used. P values <0.05, <0.01, and <0.001 were considered statistically significant (*, **, ***).

## Results

### 1,25(OH)_2_D deficiency accelerates the development of age-related spontaneous knee OA

**Figure 1:**
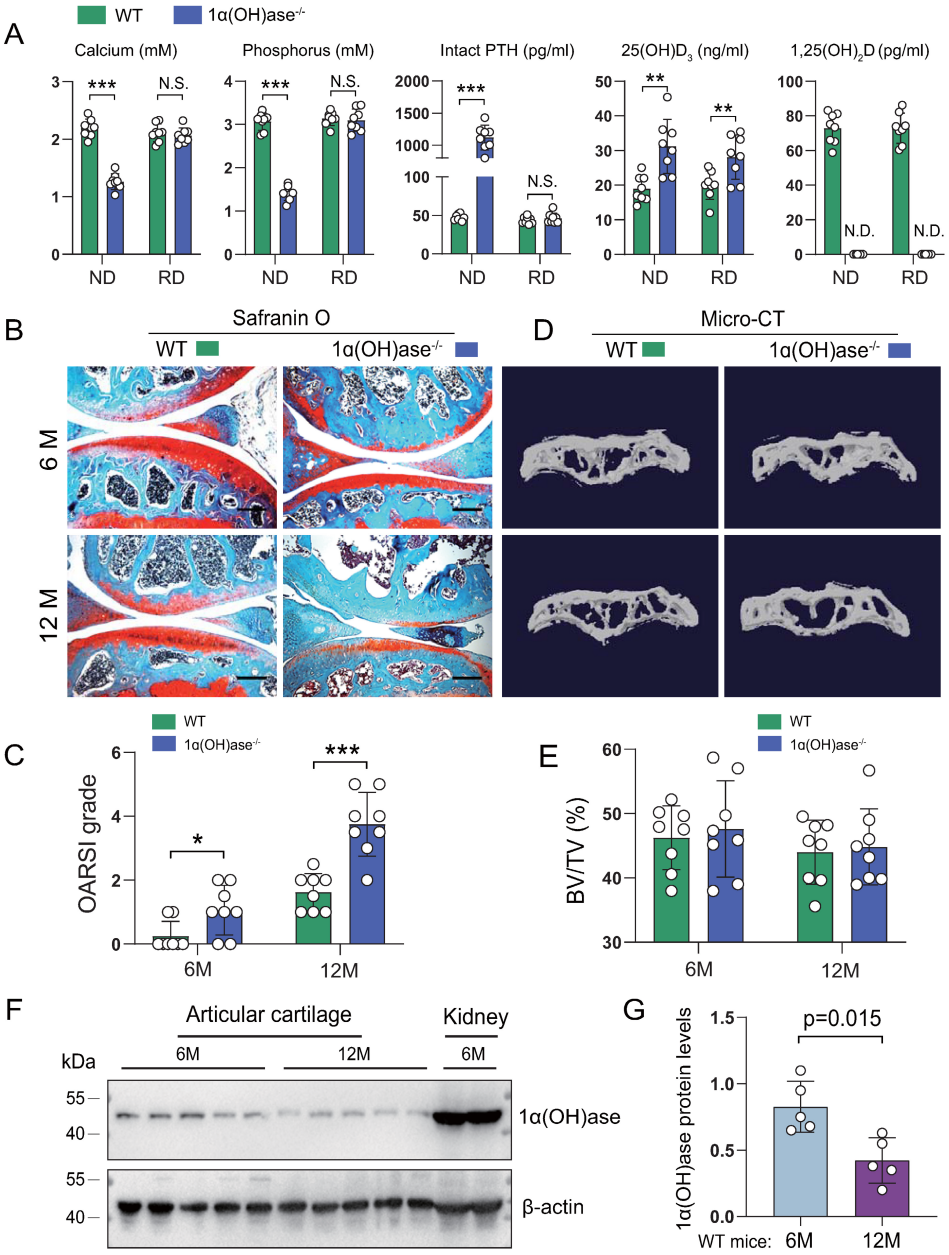
** 1,25(OH)_2_D deficiency accelerates the development of age-related spontaneous knee OA. (A)** Serum calcium, phosphorus, intact PTH, 25(OH)D and 1,25(OH)_2_D_3_ levels in 12-month-old wild-type (n=8) and 1α(OH)ase^-/-^ (n=8) mice on a normal diet (ND) or a rescue diet (RD). N.S.= not significant; N.D.= not detected. **: p< 0.01, ***: p<0.001. Representative sectioned images of articular cartilage stained for **(B)** safranin O and **(C)** the quantification of OARSI grade in 6- and 12-month-old WT and 1α(OH)ase^-/-^ mice on the rescue diet. n=8 mice per group. **(D)** Representative μCT images of knee joints and **(E)** the quantification of subchondral bone volume (BV/TV, %) in indicated groups of mice.** (F)** Western blot detection of 1α(OH)ase in articular cartilage isolated from 6- and 12-month-old WT mice (n=5 mice in each group). Kidney tissue from 6-month-old WT mice (n=2) were used as a positive control. **(G)** Quantification of 1α(OH)ase relative protein levels. *: p< 0.05, **: p< 0.01, ***: p<0.001.

To determine whether 1,25(OH)_2_D deficiency accelerated age-related knee OA, we compared the articular cartilage phenotype of 6- and 12-month-old wild-type and 1,25(OH)_2_D deficient [1α(OH)ase^-/-^] mice fed a high calcium/phosphate (rescue) diet using safranin O staining. The rescue diet normalized serum calcium, phosphorus and intact PTH levels, but did not alter increased 25(OH)D levels, while 1,25(OH)_2_D was undetectable in 1α(OH)ase^-/-^ mice (Figure 1A). Three-month-old 1α(OH)ase^-/-^ mice on the normal diet were 28% lighter than wild-type littermates (the average lifespan of 1α(OH)ase^-/-^ mice on a normal diet was 3 months) (Figure S1A), while 6- and 12-month-old 1α(OH)ase^-/-^ mice on the rescue diet were 10% and 12% lighter than control littermates, respectively. (Figure S1B). The cartilage superficial destruction, cartilage erosion, proteoglycan loss and cytopenia increased progressively with increasing age in both wild-type mice and 1α(OH)ase^-/-^ mice on the rescue diet; however, these alterations were more dramatic in 1α(OH)ase^-/-^ mice on the rescue diet compared with age-matched wild-type mice (Figure 1B). We then assessed the OARSI grade based on the sections stained with Safranin O/Fast green using the OARSI histological scoring system. The OARSI scores were progressively elevated with increasing age in both wild-type mice and 1α(OH)ase^-/-^ mice on the rescue diet, but were significantly higher in 1α(OH)ase^-/-^ mice than in age-matched wild-type mice (Figure 1C). Our previous study reported that 1,25(OH)_2_D deficiency induced aging-related bone loss 31. To test this indirect effect induced by 1,25(OH)_2_D deficiency, we examined the subchondral bone volume using micro-CT, and found that subchondral bone volume was not significantly altered in 6- and 12-month-old 1,25(OH)_2_D-deficient mice as compared with wild-type controls (Fig. 1D, E). To determine whether the age-related OA-like phenotype occurring in wild-type mice was associated with down-regulation of 1α(OH)ase in articular cartilage, we examined the protein expression level of 1α(OH)ase in articular cartilage isolated from 6- and 12-month-old wild-type mice and found that the protein levels of 1α(OH)ase in articular cartilage were significantly down-regulated in 12-month-old wild-type mice compared with 6-month-old wild-type mice (Fig. 1F, G). These results demonstrate that the enzyme which generates active 1,25(OH)_2_D declines in articular cartilage with age and that 1,25(OH)_2_D deficiency accelerates the age-related development of knee OA.

### Supplementation with 1,25(OH)_2_D_3_ rescues the knee OA phenotype in 1,25(OH)_2_D-deficient mice

**Figure 2:**
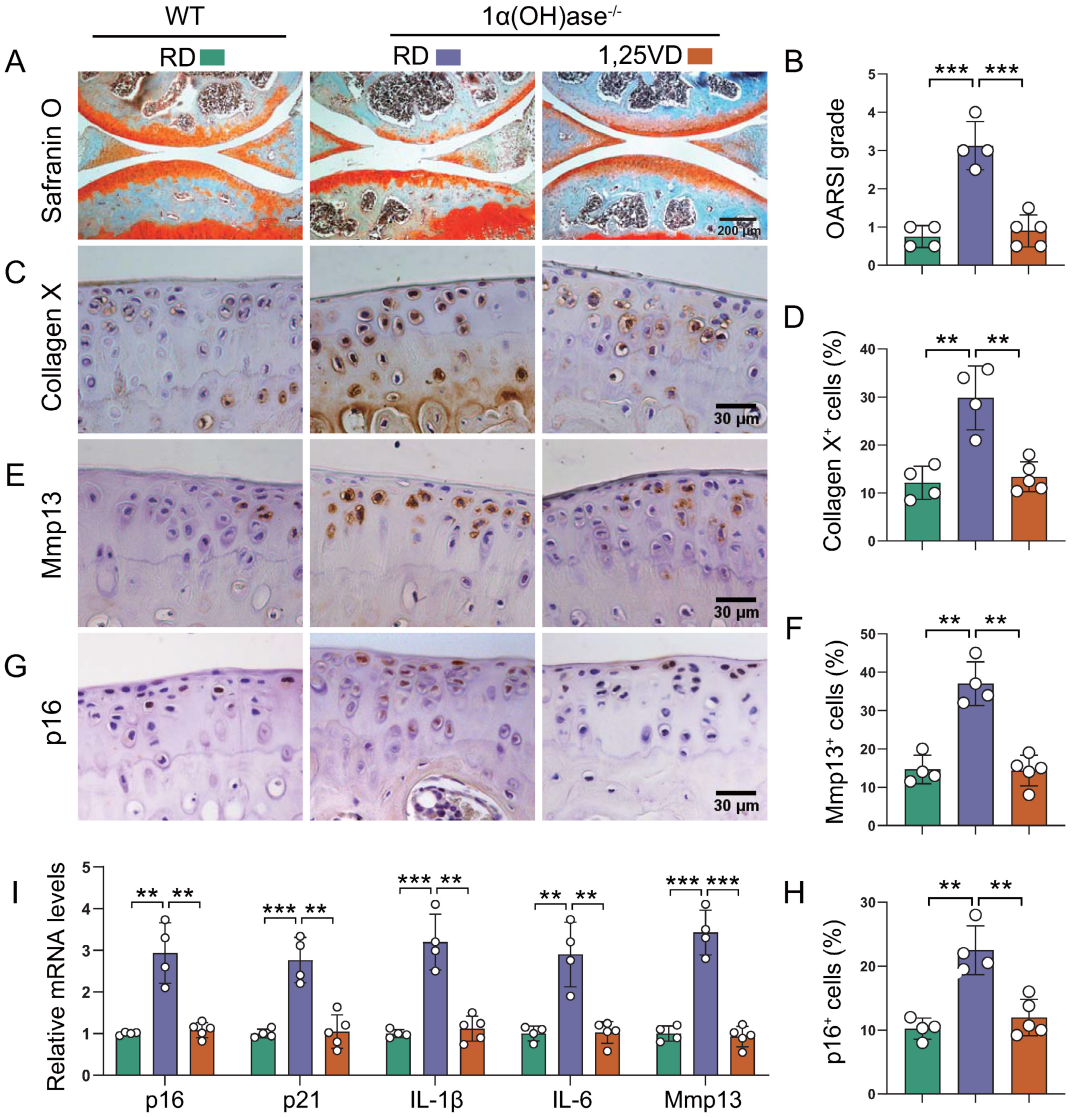
** Supplementation of 1,25(OH)_2_D_3_ rescues the knee OA phenotype caused by 1,25(OH)_2_D deficiency.** Representative images of articular cartilage stained for** (A)** safranin O and immunostained for **(C)** collagen X, **(E)** Mmp13 and **(G)** p16 in 12-month-old wild-type mice on the rescue diet (RD) (n=4), 1α(OH)ase^-/-^ mice on the RD (n=4), and 1α(OH)ase^-/-^ mice treated with 1,25(OH)_2_D_3_ (n=5). Quantification of **(B)** OARSI grade, **(D)** collagen X^+^ cells, **(F)** Mmp13^+^ cells and **(H)** p16^+^ cells. **(I)** Quantification of mRNA levels for SASP including *p16*, *p21*, *IL-1β*, *IL-6* and *Mmp13*. **: p< 0.01, ***: p<0.001.

To assess whether supplementation of exogenous 1,25(OH)_2_D_3_ can prevent knee OA caused by 1,25(OH)_2_D deficiency, 1α(OH)ase^-/-^ mice were either fed the rescue diet after weaning or were injected with 1,25(OH)_2_D_3_ subcutaneously (1 μg/kg, three times a week) after weaning until 12 months of age; wild-type mice were fed the rescue diet as the control. Serum calcium, phosphorus, and PTH levels were normalized in 1α(OH)ase^-/-^ mice fed on the rescue diet 33. Knee phenotypes were analyzed in 12-month-old mice. The cartilage superficial destruction, cartilage erosion, proteoglycan loss and cytopenia in 1α(OH)ase^-/-^ mice were largely rescued by supplementation of exogenous 1,25(OH)_2_D_3_ (Figure 2A). OARSI scores, the percentages of collagen Ⅹ^+^, Mmp13^+^, and p16^+^ articular chondrocytes, and the mRNA levels of *p16*, *p21*, *IL-1β*, *IL-6* and *Mmp13* were all significantly increased in 1α(OH)ase^-/-^ mice, and were reduced by supplementation of exogenous 1,25(OH)_2_D_3_ (Figure 2B-I). These results suggest that supplementation with exogenous 1,25(OH)_2_D_3_ rescued knee OA phenotype induced by 1,25(OH)_2_D deficiency by inhibiting chondrocyte senescence and SASP.

### 1,25(OH)_2_D_3_ rescues the reduction of human chondrocyte proliferation and increases of oxidative stress and cellular senescence induced by IL-1β

**Figure 3:**
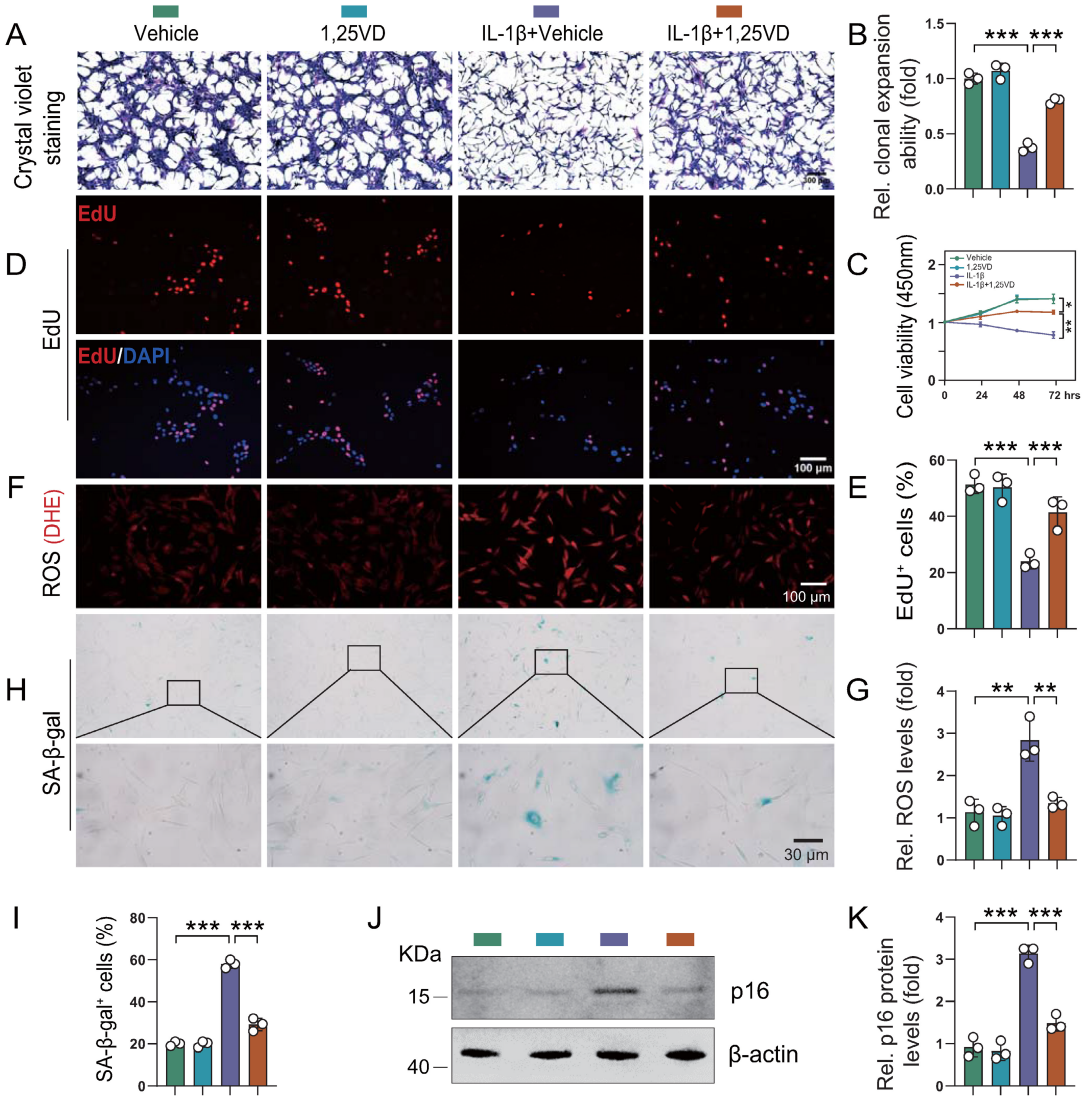
** 1,25(OH)_2_D_3_ rescues the reduction of human chondrocyte proliferation and the increases of oxidative stress and cellular senescence induced by IL-1β.** Human articular chondrocytes were cultured *in vitro* in the presence or absence of IL-1β with or without 1,25(OH)_2_D_3_ and stained **(A)** with crystal violet, **(D)** immunocytochemically for EdU, **(F)** with DHE and **(H)** cytochemically for SA-β-gal. n = 3 wells per condition. **(B)** Clonal expansion analysis. **(C)** CCK8 assay showing cell viability. **(E)** EdU-positive cells. **(G)** ROS relative levels (relative intensity of DHE fluorescence analyzed by Image J). **(I)** SA-β-gal positive cells. **(J)** Western blot detection of the cellular senescence marker p16 and **(K)** the quantification of protein levels of p16. n = 3 wells per condition. *: p< 0.05, **: p< 0.01, ***: p<0.001.

To determine the potential mechanism underlying the protective effects of 1,25(OH)_2_D_3_ on knee OA phenotypes, we studied the impact of *in vitro* application of IL-1β, a critical factor in the onset of OA 35-37. Human articular chondrocytes were cultured *in vitro* in the presence or absence of IL-1β with or without 1,25(OH)_2_D_3_ and then cell proliferation, senescence and reactive oxygen species (ROS) levels were analyzed. ROS regulate cellular homeostasis and act as prime modulators of cellular dysfunction contributing to disease pathophysiology. We firstly found that the protein expression of VDR was significantly reduced in human articular chondrocytes treated with IL-1β, and this reduction was markedly rescued upon 1,25(OH)_2_D_3_ treatment (Figure S2A-B). In addition, results revealed that the density and viability of chondrocytes and percentage of EdU-positive chondrocytes were significantly reduced in IL-1β + vehicle-treated cultures relative to vehicle-treated cultures, whereas they were insignificantly altered in 1,25(OH)_2_D_3_-treated cultures relative to vehicle-treated cultures. However, EdU-positive chondrocytes were significantly increased in IL-1β + 1,25(OH)_2_D_3_-treated cultures relative to IL-1β + vehicle-treated cultures (Figure 3A-E). Furthermore, IL-1β was reported to induce an OA cell phenotype *in vitro*, that includes increased oxidative stress and cellular senescence 35. Here we found that ROS levels, the percentage of SA-β-gal positive chondrocytes and protein levels of p16 were significantly increased in IL-1β + vehicle-treated cultures relative to vehicle-treated cultures, whereas they were insignificantly altered in 1,25(OH)_2_D_3_-treated cultures relative to vehicle-treated cultures. However, these parameters of oxidative stress and cellular senescence were significantly decreased in IL-1β + 1,25(OH)_2_D_3_-treated cultures relative to IL-1β + vehicle-treated cultures (Figure 3F-K). These results demonstrated that 1,25(OH)_2_D_3_ could rescue the reduction of chondrocyte proliferation and the increase of oxidative stress and cellular senescence induced by IL-1β.

### VDR deficiency induces chondrocyte senescence and a knee OA phenotype

**Figure 4:**
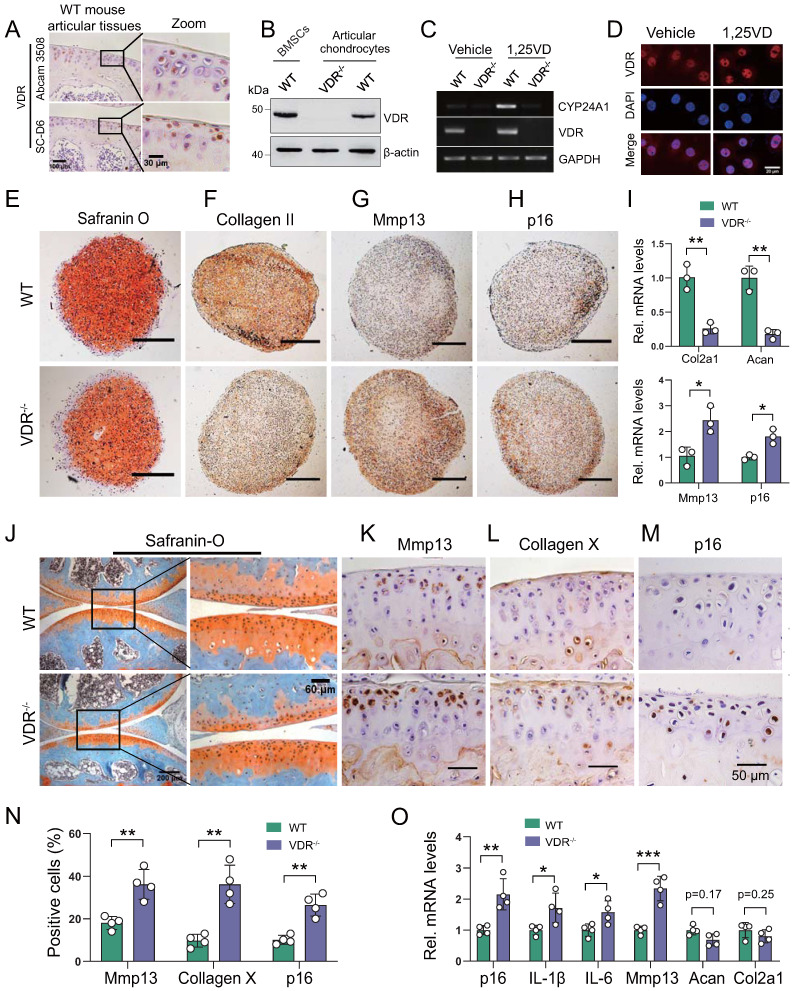
** VDR deficiency induces chondrocyte senescence and a knee OA phenotype. (A)** Representative micrographs of articular cartilage sections of 6-month-old wild-type mice (n=5) immunostained for VDR using two sources of VDR antibody (Abcam 3508 and Santa Cluz D6). **(B)** Western blot detection of VDR in monolayer-cultured wild-type mouse chondrocytes. Proteins isolated from wild-type BM-BMSCs and VDR^-/-^ chondrocytes were loaded as positive and negative control, respectively. **(C)** Marked increase of *Cyp24a1* and *VDR* mRNA levels in 1,25(OH)_2_D_3_-treated wild-type mouse chondrocytes, but not in VDR^-/-^ cells demonstrated by RT-PCR. GAPDH was used as a loading control. **(D)** Immunofluorescence staining for VDR in mouse chondrocytes after treatment with vehicle or 1,25(OH)_2_D_3_ for 24 h. 1,25(OH)_2_D_3_-treated cells showed an increased localization of VDR in the nucleus compared with vehicle-treated cells. **(E-H)** Representative images of sections from wild-type (n=3) and VDR^-/-^ (n=3) chondrocyte micromass cultures stained with (E) safranin O and immunostained for (F) collagen II, (G) Mmp13 and (H) p16 (scale bars, 200 µm). **(I)** Relative mRNA levels of *col2a1, aggrecan*, *Mmp13* and *p16* in macromass cultures above. n=3 wells per group. Representative micrographs of sections from 6-month-old wild-type (n=4) and VDR^-/-^ (n=4) mice on the rescue diet stained **(J)** with safranin O and immunostained for **(K)** Mmp13, **(L)** collagen X and **(M)** p16. **(N)** Statistical analysis of Mmp13^+^, collagen X^+^ and p16^+^ cells. **(O)** Relative mRNA levels of *p16*, *IL-1β*, *IL-6*, *Mmp13*, *aggrecan* and *col2a1* in wild-type and VDR^-/-^ cartilage. *: p< 0.05, **: p< 0.01, ***: p<0.001.

We next assessed whether the development of knee OA induced by 1,25(OH)_2_D deficiency was mediated through VDR. We firstly confirmed the protein expression of VDR in articular chondrocytes by immunohistochemistry and Western blots in WT mouse articular tissues (Figure 4A) and in BMSCs (Figure 4B). To determine whether VDR in cartilage chondrocytes can respond to exogenous 1,25(OH)_2_D_3_, articular chondrocytes were treated with 10^-8^ M 1,25(OH)_2_D_3_, and the mRNA expression levels of VDR and CYP24A1, a known downstream target gene of VDR, were examined using RT-PCR; nuclear localization of VDR was also examined by immunofluorescence. The results showed that the mRNA levels of VDR and CYP24A1 were clearly increased, and VDR nuclear localization was also clearly increased in wild-type chondrocytes, but not in VDR^-/-^ cells when they were treated with 1,25(OH)_2_D_3_ (Figure 4C, D). To determine whether VDR deficiency induces chondrocyte senescence and a knee OA phenotype, the phenotypes of micromass pellets from micromass cultures of articular chondrocytes derived from wild-type and VDR^-/-^ mice, and the knee phenotypes from 6-month-old wild-type and VDR^-/-^ mice on the rescue diet were analyzed. We found that serum calcium, phosphorus, and PTH levels were normalized in VDR^-/-^ mice on the rescue diet relative to wild-type mice (Figs. S3A-C); however, safranin O and type II collagen, and the mRNA expression levels of *col2a1* and *aggrecan* were decreased, whereas Mmp13^+^ and p16^+^ chondrocytes and their mRNA expression levels were increased in VDR^-/-^ articular chondrocytes compared with wild-type ones (Figure 4E-I). Cartilage erosion, proteoglycan loss and cytopenia were observed in VDR^-/-^ mice relative to their wild-type littermates (Figure 4J). The percentages of Mmp13^+^, Collagen Ⅹ^+^, and p16^+^ chondrocytes, and the mRNA expression levels of *p16*, *IL-1β*, *IL-6*, *Mmp13*, were all significantly increased, whereas the mRNA expression levels of *aggrecan* and *col2a1* were markedly decreased in VDR^-/-^ mice relative to their wild-type littermates (Figure 4K-O). These results demonstrated that VDR deficiency also induced knee OA by stimulating chondrocyte senescence and SASP, indicating that the development of knee OA induced by 1,25(OH)_2_D was mediated though VDR.

### 1,25(OH)_2_D_3_ suppresses IL-1β-induced chondrocyte senescence by VDR-mediated transcriptional up-regulation of Sirt1

**Figure 5:**
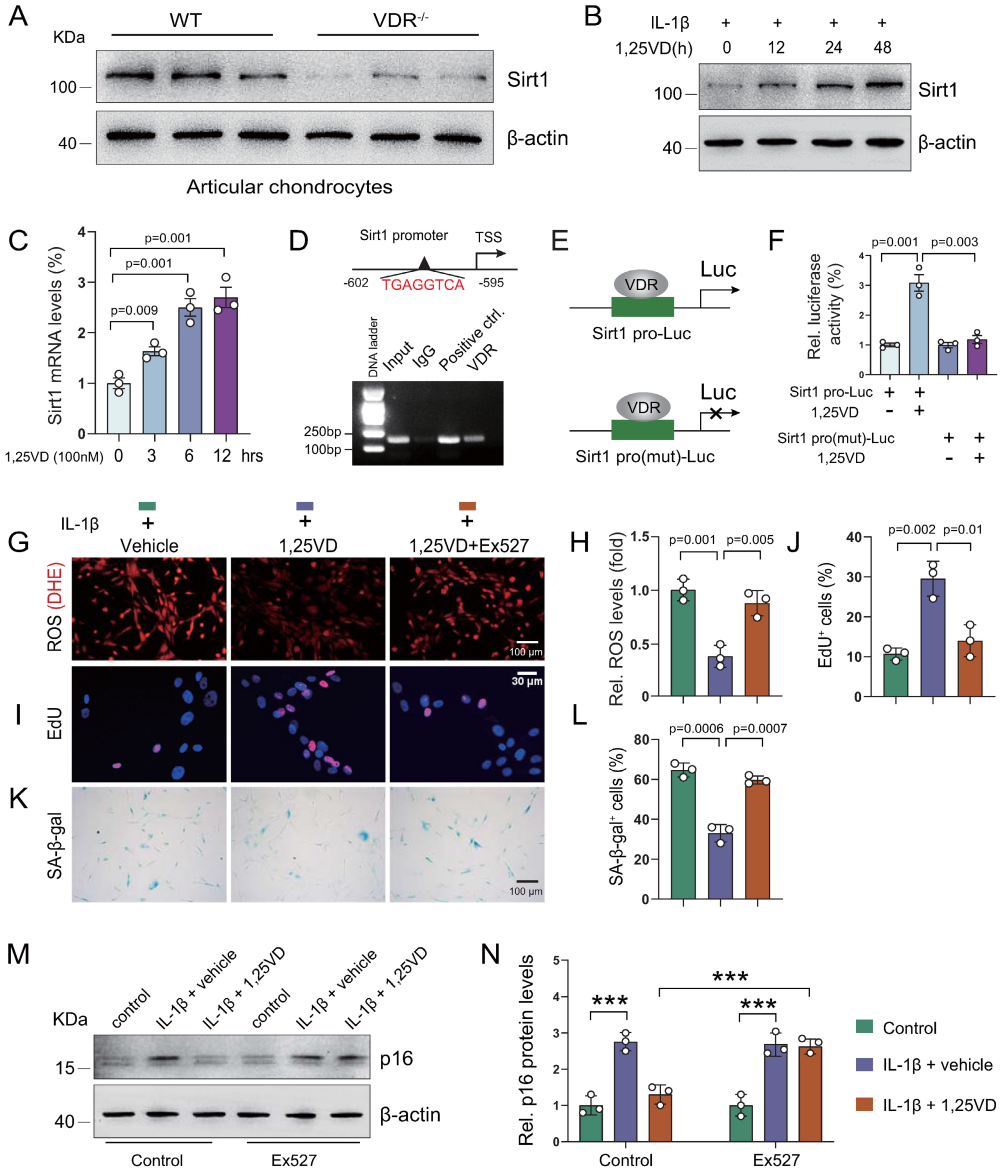
** 1,25(OH)_2_D_3_ suppresses IL-1β-induced chondrocyte senescence by VDR-mediated transcriptional up-regulation of Sirt1. (A)** Western blot detection of Sirt1 in articular chondrocytes isolated from WT (n=3) and VDR^-/-^ (n=3) mice. **(B)** Western blot detection of Sirt1 in human articular chondrocyte cultures treated with IL-1β alone or combined with 1,25(OH)_2_D_3_ for indicated times. **(C)** Relative Sirt1 mRNA levels in human articular chondrocytes cultured with 1,25(OH)_2_D_3_ for 3, 6 and 12 hours. **(D)** Upper: VDRE-like elements in human Sirt1 promoter region highlighted in red. Lower: Chromatin immunoprecipitation (ChIP) with IgG antibody, H3 antibody (positive control) or VDR antibody were performed in human articular chondrocytes and relative enrichment of human Sirt1 promoter was determined using RT-PCR assay. **(E)** Human Sirt1 promoter or Sirt1 promoter mutant Luc-plasmid were transfected into human articular chondrocytes followed by vehicle or 1,25(OH)_2_D_3_ treatment for 12 hours and **(F)** relative luciferase activity was analyzed after 48 hours. n=3 replicates per condition. Human articular chondrocytes were cultured with vehicle, 1,25(OH)_2_D_3_, or 1,25(OH)_2_D_3_ plus Ex527 (a Sirt1 inhibitor) in the presence of IL-1β and stained **(G)** with dihydroethidium (DHE) to determine relative ROS levels, **(I)** cytochemically for EdU and** (K)** cytochemically for SA-β-gal. n=3 replicates per condition. Statistical analysis of **(H)** DHE fluorescence intensity, **(J)** EdU-positive cells and **(L)** SA-β-gal-positive cells. **(M)** Protein expression levels of p16 in human articular chondrocytes treated with vehicle or the Sirt1 inhibitor (Ex527) in the presence or absence of IL-1β and 1,25(OH)_2_D_3_. n=3 wells per condition. **(N)** Quantification of (M). *: p< 0.05, ***: p<0.001.

To uncover the molecular mechanism underlying how 1,25(OH)_2_D_3_ inhibits IL-1β-induced oxidative stress and cellular senescence, we treated human articular chondrocytes with vehicle or 1,25(OH)_2_D_3_ in the presence of IL-1β for 72 hours; subsequent KEGG pathways enrichment results showed that 1,25(OH)_2_D_3_ significantly regulated pathways such as “Cellular senescence” and “FoxO signaling pathway”, which are target pathways of Sirt1 (Figure S4A). To determine whether 1,25(OH)_2_D_3_ suppressed IL-1β-induced chondrocyte senescence by transcriptionally up-regulating Sirt1 in a VDR dependent manner, we examined the expression of Sirt1 in articular cartilage of WT and 1α(OH)ase^-/-^ mice with and without applications of supplementary 1,25(OH)_2_D_3_. We found that exogenous 1,25(OH)_2_D_3_ rescued 1,25(OH)_2_D deficiency-induced reduction of Sirt1-positive chondrocytes (Figure S4B, C). We also found that the protein expression level of Sirt1 was significantly down-regulated in articular chondrocytes isolated from VDR^-/-^ mice relative to those isolated from wild-type mice (Figure 5A). Human articular chondrocytes were treated with vehicle- and 1,25(OH)_2_D_3_ at indicated time points (0, 12, 24, and 48 hours for protein analyses, or 0, 3, 6 and 12 hours for mRNA analyses) in the presence of IL-1β. The results showed that 1,25(OH)_2_D_3_ up-regulated the protein and mRNA expression of Sirt1 in a time dependent manner (Figure 5B & C). To further determine whether 1,25(OH)_2_D_3_ regulates Sirt1 via the VDR at a transcriptional level, a VDRE-like sequence located upstream of the Sirt1 gene (http://jaspar.genereg.net/) was identified (Figure 5D, upper). The ChIP-PCR assay showed that VDR could directly bind to the Sirt1 promoter at the predicted binding site (Figure 5D, lower). Luciferase reporter assays showed that 1,25(OH)_2_D_3_ increased luciferase activity significantly in human articular chondrocytes transfected with a Sirt1 pro-Luc plasmid, but failed to activate the pro-mutant Luc reporter (Figure 5E & F). Furthermore, we found that 1,25(OH)_2_D_3_ inhibited ROS accumulation, as determined by dihydroethidium (DHE) staining (Figure 5G & H), increased chondrocyte proliferation (Figure 5I & J), decreased chondrocyte senescence (Figure 5K & L) and up-regulated p16 protein expression (Figure 5M & N) in human articular chondrocyte treated with IL-1β, and these parameters were largely blocked by treatment with the Sirt1 inhibitor, Ex527 (Figure 5G-N). These results demonstrated that 1,25(OH)_2_D_3_ suppresses IL-1β-induced chondrocyte senescence by VDR-mediated transcriptional up-regulation of Sirt1.

### Overexpression of Sirt1 in MSCs prevents 1,25(OH)_2_D deficiency-induced development of knee OA

**Figure 6:**
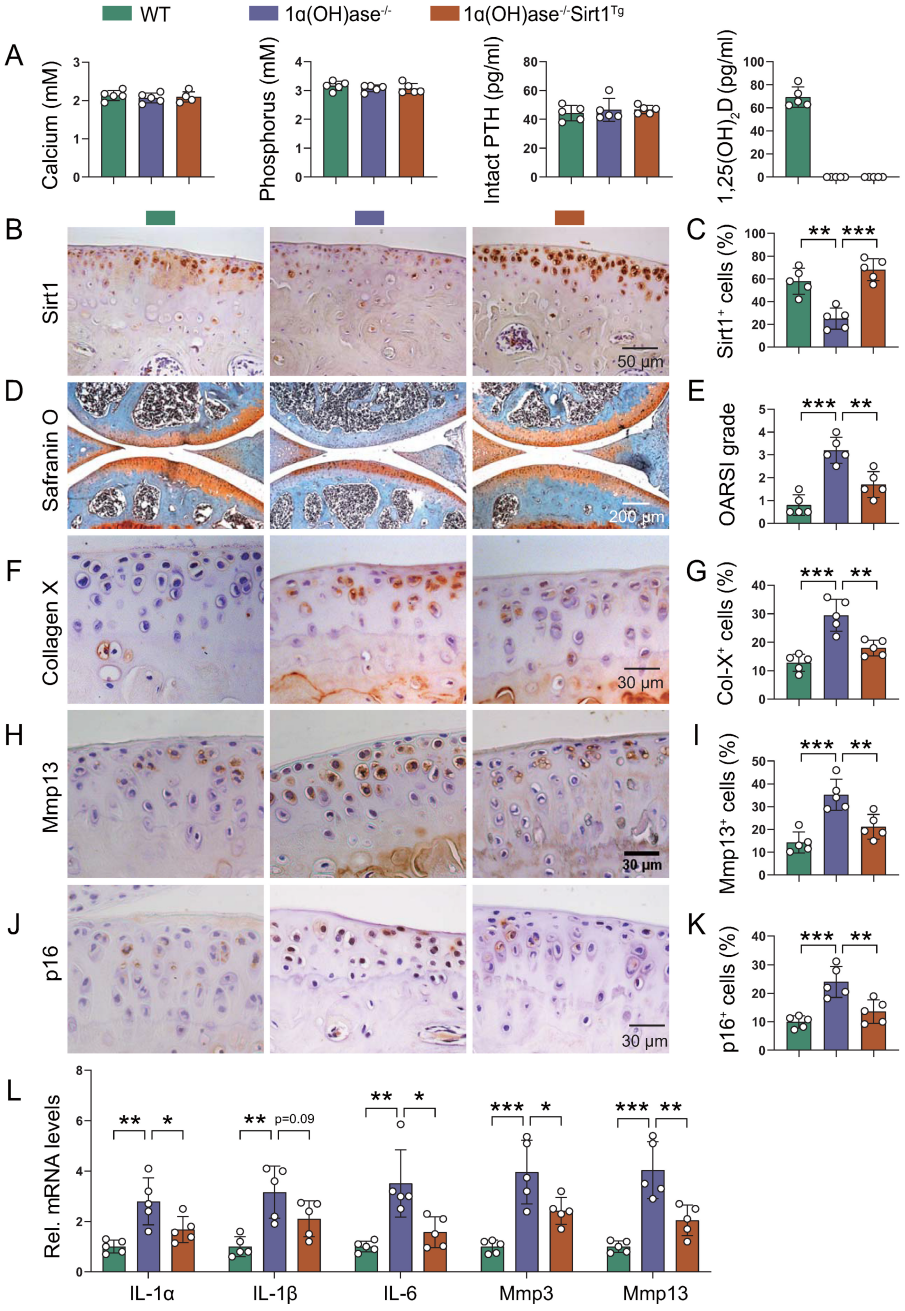
** Overexpression of Sirt1 in MSCs prevents 1,25(OH)_2_D deficiency-induced development of knee OA. (A)** Serum calcium, phosphorus, intact PTH and 1,25(OH)_2_D_3_ levels in 12-month-old wild-type (n=5), 1ɑ(OH)ase^-/-^ (n=5) and 1ɑ(OH)ase^-/-^Sirt1^Tg^ mice (n=5) on the rescue diet (RD). **(B)** Representative micrographs of articular cartilage sections immunostained for Sirt1 in indicated groups of mice and **(C)** a quantitative analysis of the percentage of Sirt1-positive chondrocytes. Representative micrographs of knee sections stained with **(D)** safranin O, and immunostained for **(F)** collagen X, **(H)** Mmp13 and **(J)** p16. Evaluation for **(E)** OARSI score and quantification of **(G)** collagen X^+^, **(I)** Mmp13^+^ and **(K)** p16^+^ cells. **(L)** Relative mRNA levels of SASP factors including *IL-1ɑ, IL-1β, IL-6, Mmp3, Mmp13* were analyzed using qPCR. n=5 mice per group. *: p< 0.05, **: p< 0.01, ***: p<0.001.

Due to the complexity and heterogeneity of knee OA pathogenesis, the combination of the removal of prevalent senescent cells and the rejuvenation of MSCs has been established as a potential therapeutic option 21-23, 38. To assess whether overexpression of Sirt1 in MSCs could prevent 1,25(OH)_2_D deficiency-induced development of knee OA, we crossed Prx1-Sirt1 transgenic mice with 1α(OH)ase^-/-^ mice to generate 1α(OH)ase^-/-^ mice with Sirt1 overexpression in MSCs [1α(OH)ase^-/-^ Sirt1^Tg^], and compared their knee phenotypes with 1α(OH)ase^-/-^ mice on the rescue diet and wild-type littermates. Serum calcium, phosphorus, and PTH levels were normalized, whereas serum 1,25(OH)_2_D_3_ was undetectable in both 1α(OH)ase^-/-^ and 1α(OH)ase^-/-^ Sirt1^Tg^ mice (Figure 6A). In addition, the percentage of Sirt1-positive chondrocytes was significantly increased in 1α(OH)ase^-/-^ Sirt1^Tg^ mice as compared with WT and 1α(OH)ase^-/-^ mice (Figure 6B & C). The overexpression of Sirt1 in MSCs rescued all knee OA phenotypes observed in 12-month-old 1α(OH)ase^-/-^ mice, including the significantly increased OARSI scores (Figure 6D & E), the increased percentages of collagen Ⅹ^+^ (Figure 6F & G), Mmp13^+^ (Figure 6H & I), and p16^+^ (Figure 6J & K) chondrocytes, and the increased mRNA levels of *IL-1α*, *IL-1β*, *IL-6*, *Mmp3*, and *Mmp13* (Figure 6L). These results demonstrated that overexpression of Sirt1 in MSCs could partially prevent the development of 1,25(OH)_2_D deficiency-induced knee OA by inhibiting chondrocyte senescence and SASP.

## Discussion

The cause of aging-related OA is likely complex and the direct roles of VDR and 1,25(OH)_2_D in OA are largely unknown or controversial. In this study we demonstrated, for the first time, a direct link between the 1,25(OH)_2_D-VDR signaling pathway and the onset of age-related OA at tissue-, cellular-, and molecular-levels using multiple genetically-modified mouse lines and comprehensive analytical techniques, including histopathology, 3D cultures of human and mouse chondrocytes, immunofluorescence and immunohistochemistry staining, chromatin immunoprecipitation and dual-luciferase assay. Our key findings are 1) 1,25(OH)_2_D deficiency accelerated the development of age-related spontaneous knee OA; 2) 1,25(OH)_2_D_3_ supplementation rescued all knee OA phenotypes of 1α(OH)ase^-/-^ mice *in vivo*, and 1,25(OH)_2_D_3_ rescued IL-1β-induced chondrocyte OA phenotypes *in vitro*; 3) VDR knockout mice exhibited knee OA phenotypes; 4) 1,25(OH)_2_D_3_ up-regulated Sirt1 through VDR mediated transcription in human articular chondrocytes; 5) Overexpression of Sirt1 in MSCs rescued knee OA phenotypes in 1α(OH)ase^-/-^ mice. Our results indicate that age-related knee OA induced by 1,25(OH)_2_D deficiency occurs in a calcium-, phosphorus- and 25(OH)D-independent manner. Our results may help to explain the inconsistent results surrounding the association between vitamin D levels, as measured by serum 25(OH)D, and OA. Serum 25(OH)D levels are not decreased in 1α(OH)ase^-/-^ mice relative to wild-type mice 12; however, our previous studies demonstrated that 1α(OH)ase^-/-^ mice display female infertility, male infertility, hypertension, premature aging, a high incidence of multiple tumors and osteoporosis 31, 33, 39-44. These results suggest that sufficient 1,25(OH)_2_D rather than 25(OH)D levels are necessary for the prevention of vitamin D deficiency-induced diseases including knee OA.

**Figure 7:**
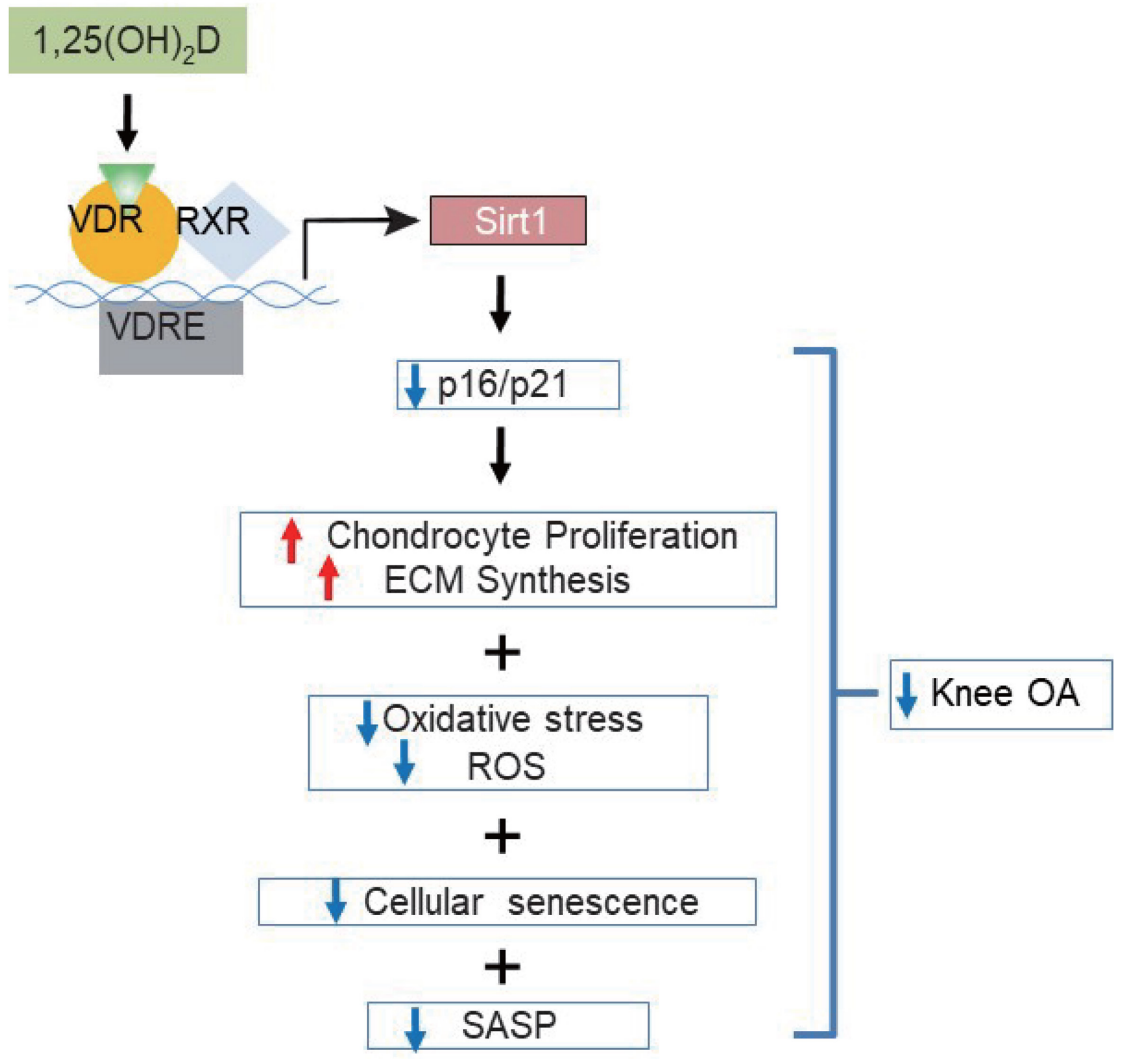
Model of mechanisms leading from 1,25(OH)_2_D_3_ to prevent age-related spontaneous knee osteoarthritis via VDR-mediated upregulation of Sirt1.

Vitamin D supplementation may improve pain and function in patients with knee OA; however, the evidence from observational studies and randomized controlled trials are controversial 45, 46. To determine whether supplementation of exogenous 1,25(OH)_2_D_3_ can prevent knee OA caused by 1,25(OH)_2_D deficiency, we performed *in vivo* supplementation of exogenous 1,25(OH)_2_D_3_ in 1α(OH)ase^-/-^ mice and *in vitro* treatment of 1,25(OH)_2_D_3_ in 2D or 3D cultures of human articular chondrocytes with IL-1β induction. Our *in vivo* experiments revealed that all knee OA phenotypes observed in 1α(OH)ase^-/-^ mice were rescued by supplementation with exogenous 1,25(OH)_2_D_3_. *In vitro* 2D models have used cytokines such as IL-1β to induce an OA phenotype which has allowed for the screening of chondroprotective compounds to attenuate the catabolic factors involved in articular chondrocyte degradation 47. Both healthy and OA articular chondrocytes express the IL-1 receptor type I. The effect of IL-1β on chondrocytes can be generally described as catabolic and involves the upregulation of aggrecanases and MMPs, the induction of additional inflammatory mediators, as well as the downregulation of chondrogenic extracellular matrix synthesis 48. A recent study implies that omentin-1 could have clinical relevance in the treatment of osteoarthritis by inhibiting IL-1β-induced chondrocyte senescence 49. In our *in vitro* experiments, we found that IL-1β inhibited the proliferation of human articular chondrocytes, and stimulated oxidative stress and articular chondrocyte senescence. Treatment with 1,25(OH)_2_D_3_, but not with vehicle, rescued IL-1β-induced chondrocyte phenotypes. Consequently, supplementation with exogenous 1,25(OH)_2_D_3_ prevented the onset and progression of knee OA by stimulating chondrocyte proliferation and extracellular matrix synthesis, and by inhibiting chondrocyte differentiation and degradation of extracellular matrix proteins and chondrocyte senescence in articular cartilage (Figure 7). In a recent study, we reported that progressive down-regulation of the protein expression levels of 1α(OH)ase in kidney, intestine and bone of 3-, 9- and 18-month-old wild-type mice were associated with progressive bone loss 30. In the current study, we found that the protein expression level of 1α(OH)ase in articular cartilage was also reduced in 12-month-old wild-type mice relative to 6-month-old wild-type mice. Consequently, whether vitamin D supplementation can prevent the onset and progression of knee OA may depend on the local 1α(OH)ase expression levels and activity that is required to convert supplementary vitamin D into active vitamin D.

It has been reported that VDR is expressed in the articular cartilage of OA patients and human articular chondrocytes 50. We therefore asked whether the development of knee OA induced by 1,25(OH)_2_D deficiency was mediated though VDR. To address this issue, we first confirmed that VDR is localized in mouse articular chondrocytes as demonstrated by both immunohistochemistry and Western blots. We also found that treatment with 1,25(OH)_2_D_3_ could up-regulate VDR expression and increase its nuclear translocation in human articular chondrocytes. Then we demonstrated that VDR deficiency resulted in the reduction of extracellular matrix protein synthesis and the increase of Mmp13 and p16 expression in 3D cultured mouse articular chondrocytes. VDR deficiency also induced knee OA phenotypes *in vivo* by enhancing extracellular matrix degradation and articular chondrocyte hypertrophic differentiation and stimulating chondrocyte senescence and SASP. Consequently, VDR deficiency could induce the development of knee OA, supporting the role of VDR mediated 1,25(OH)_2_D deficiency in the development of knee OA.

The expression of Sirt1 in articular cartilage is negatively associated with severity of knee OA 51, and cartilage-specific Sirt1-deficient mice develop accelerated OA progression under mechanical stress and aging 20. Our current study demonstrated that the expression level of Sirt1 was dramatically reduced in articular chondrocytes from 1,25(OH)_2_D deficient mice, and this was rescued by supplementation with exogenous 1,25(OH)_2_D_3._ Treatment with 1,25(OH)_2_D_3_ significantly upregulated the expression of Sirt1 at both protein and mRNA levels in human articular chondrocytes in the presence of IL-1β, while the inhibitory effect of 1,25(OH)_2_D_3_ on IL-1β-induced chondrocyte senescence and p16 up-regulation were largely blocked by treatment with the Sirt1 inhibitor, Ex527. Furthermore, we demonstrated that 1,25(OH)_2_D_3_ up-regulated Sirt1 expression via VDR-mediated transcription in human articular chondrocytes. Consequently, Sirt1 appears to be a direct downstream target of 1,25(OH)_2_D.

In view of the fact that previous studies suggested that Sirt1 plays a preventive role against the development of OA 18, 20, we investigated whether overexpressing Sirt1 in MSCs could prevent 1,25(OH)_2_D deficiency-induced development of knee OA. We found that similar to supplementation with 1,25(OH)_2_D_3_, overexpressing Sirt1 in MSCs rescued 1,25(OH)_2_D-deficiency-induced knee OA by up-regulating Sirt1 expression in articular chondrocytes, inhibiting the differentiation of chondrocytes, degradation of extracellular matrix proteins, chondrocyte senescence and SASP in articular cartilage (Figure 7). Therefore, our results indicate that 1,25(OH)_2_D can prevent the development of knee OA by inhibiting extracellular matrix degradation, cellular senescence and SASP via Sirt1 mediation (Figure 7).

We recently showed that 1,25(OH)_2_D deficiency accelerates age-related bone loss 31, raising the question of whether 1,25(OH)_2_D deficiency-induced OA is actually driven by the loss of subchondral bone. In this study, we found that the subchondral bone volume of 1,25(OH)_2_D deficient mice was not significantly altered compared with WT controls. In contrast, VDR-knockout chondrocytes displayed cellular senescence and an OA phenotype *in vitro*, and 1,25(OH)_2_D also protected chondrocytes against an IL-1β-induced OA phenotype *in vitro*, including increased ROS levels and cellular senescence. These findings demonstrate that 1,25(OH)_2_D can prevent an OA phenotype via VDR by directly targeting chondrocytes. However, the clarification of the role of subchondral bone in contributing to age-related OA is a potential limitation of our study, and aged mice with specific deletion of VDR in subchondral bone and in chondrocytes should be constructed to further examine the direct contribution of vitamin D deficiency in subchondral bone and in chondrocytes to an OA phenotype with aging.

Overall, the results of this study provide a model that suggests that 1,25(OH)_2_D via the VDR prevents aging-related knee OA by up-regulating Sirt1 through enhanced VDR mediated gene transcription, activating p16/p21 signaling and decreasing oxidative stress, promoting articular chondrocyte proliferation and extracellular matrix protein synthesis, and reducing articular chondrocyte senescence and SASP (Figure 7). This study therefore not only identifies novel mechanisms of 1,25(OH)_2_D deficiency in accelerating age-related knee OA development, but may also provide experimental and theoretical evidence for potential utilization of 1,25(OH)_2_D_3_ or downstream targets, including Sirt1 activators to prevent age-related knee OA.

## Supplementary Material

Supplementary figures and table.
