# Supplementary material for: 1,25-Dihydroxyvitamin D Deficiency Accelerates Aging-related Osteoarthritis via Downregulation of Sirt1 in Mice
Source: Int J Biol Sci. 2023 Jan 1;19(2):610–24. doi: 10.7150/ijbs.78785 (PMC9830508; doi:10.7150/ijbs.78785)
Supplement: Supplementary file 1 — Supplementary figures and table. [file ijbsv19p0610s1.pdf]

Supplementary materials

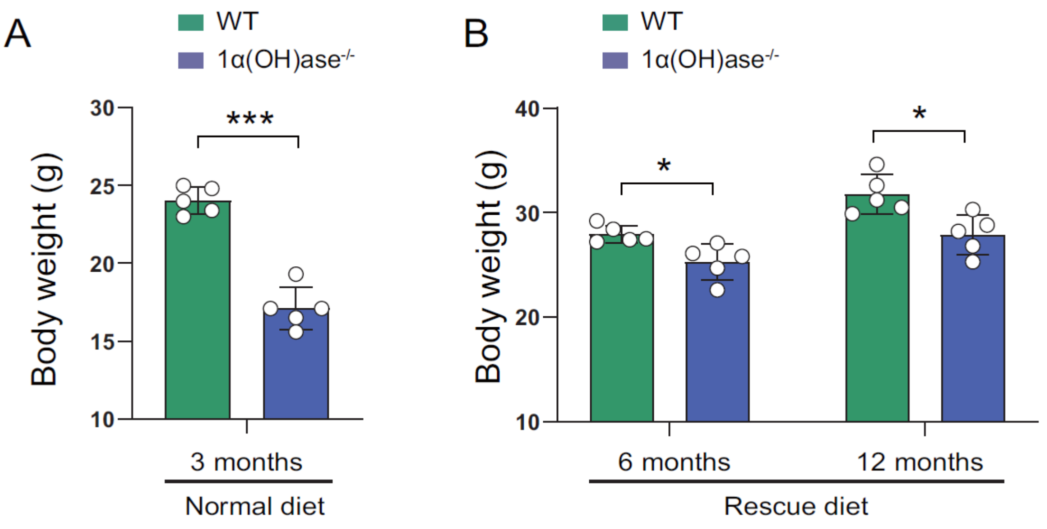

Figure S1. Body weight of WT and 1α(OH)ase$^{-/-}$ mice

(A) Body weight of 3-month-old WT and 1α(OH)ase$^{-/-}$ mice fed a normal diet. (B) Body weight of 6- and 12-month-old WT and 1α(OH)ase$^{-/-}$ mice fed a rescue diet. *: $p< 0.05$, ***: $p< 0.01$.

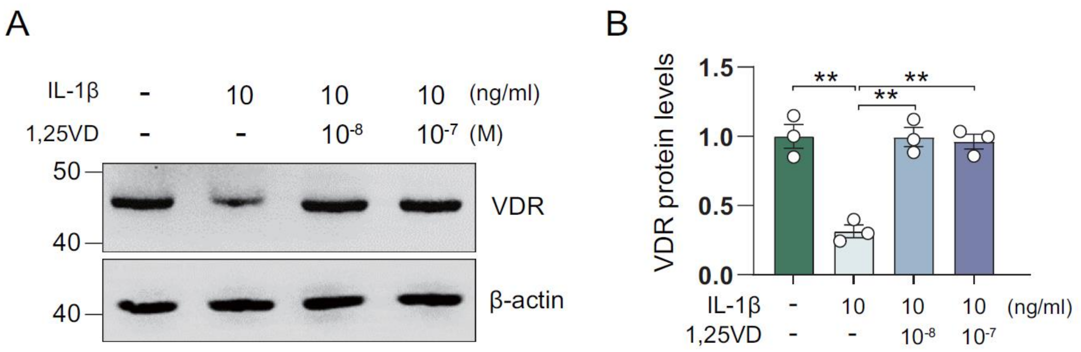

Figure S2. VDR protein levels were decreased upon IL-1β stimulation, and increased upon 1,25(OH)$_2$D$_3$ treatment in human articular chondrocytes in vitro

(A) Western blot detection of VDR in human articular chondrocytes treated with IL-1β in the presence or absence of 1,25(OH)2D3. (B) Quantification of (A). n=3 wells per condition. *: $p< 0.05$, **: $p< 0.01$.

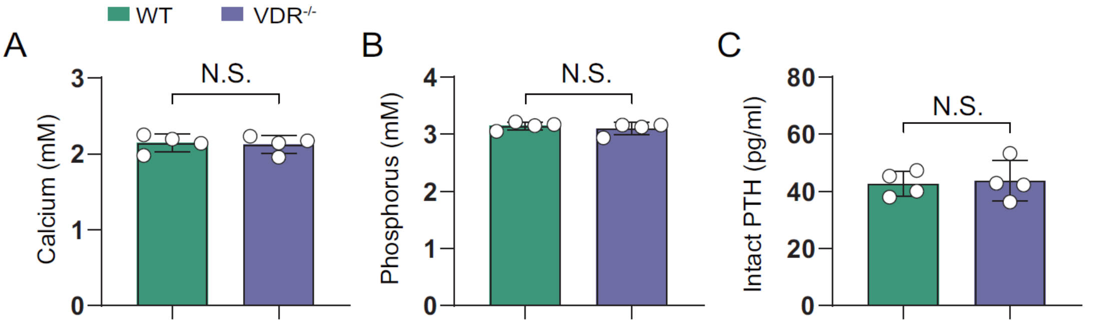

Figure S3. Serum calcium, phosphorus and intact PTH levels in WT and VDR$^{-/-}$ mice

(A) Serum calcium, (B) phosphorus and (C) intact PTH levels in 6-month-old WT (n=4) and VDR-/- mice (n=4) on the rescue diet (RD). N.S.= not significant.

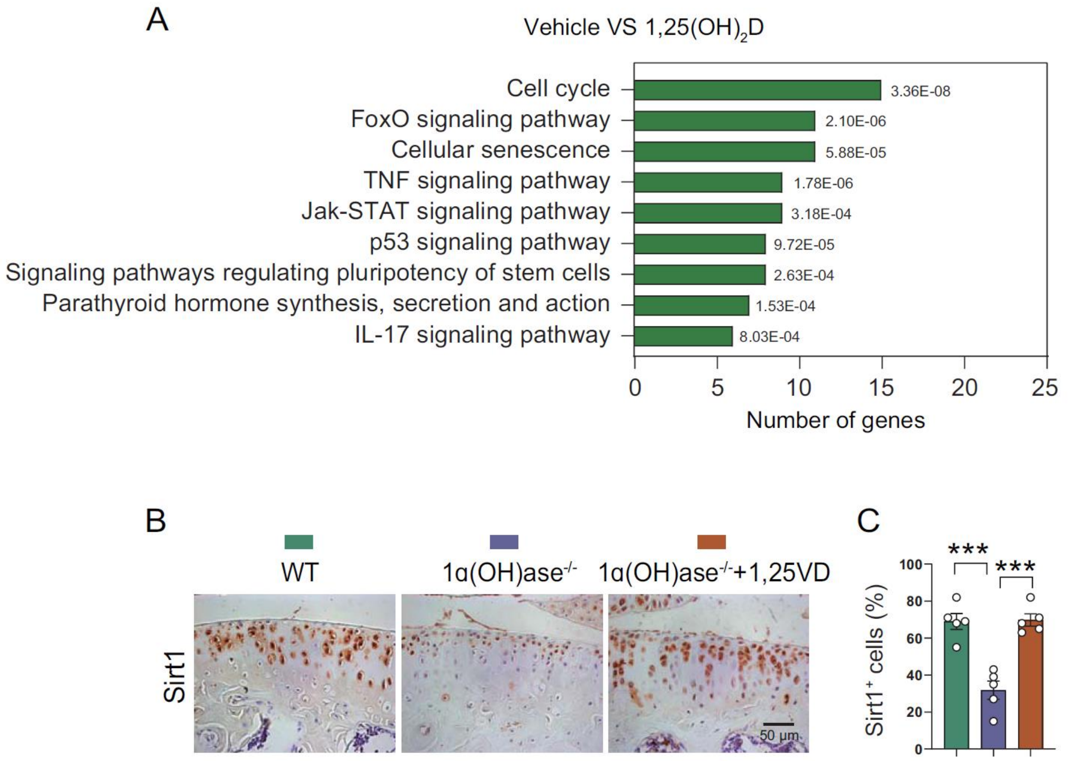

Figure S4. Differentially expressed pathways involved in $1,25(OH)_2D_3$-treated human articular chondrocytes in the presence of IL-1β

(A) KEGG enrichment analysis of pathways involved in $1,25(OH)_2D_3$-treated human articular chondrocytes in the presence of IL-1β. P-value was labeled in red. n=3 wells per condition. (B) Representative micrographs of sections from wild-type and 1α(OH)ase$^{-/-}$ mice without or with 1,25(OH)2D3 treatment were immunostained for Sirt1. n=5 mice per group. (C) Quantification of Sirt1+ cells. ***: p<0.001.

Supplementary Table 1. Primers used for quantitative real-time PCR

		Forward	Reverse
**GAPDH**	Mouse	AGGTCGGTGTGAACGGATTTG	TGTAGACCATGTAGTTGAGGTCA
**IL-6**	Mouse	GCTACCAAACTGGATATAATCAGGA	CCAGGTAGCTATGGTACTCCAGAA
**IL-1α**	Mouse	CGAAGACTACAGTTCTGCCATT	GACGTTTCAGAGGTTCTCAGAG
**IL-1β**	Mouse	GCAACTGTTCCTGAACTCAACT	ATCTTTTGGGGTCCGTCAACT
**Mmp3**	Mouse	ACATGGAGACTTTGTCCCTTTTG	ACATGGAGACTTTGTCCCTTTTG
**Mmp13**	Mouse	CTTCTTCTTGTTGAGCTGGACTC	CTGTGGAGGTCACTGTAGACT
**p16**	Mouse	GAAAGAGTTCGGGGCGTTG	GAGAGCCATCTGGAGCAGCAT
**p21**	Mouse	CCTGGTGATGTCCGACCTG	CCATGAGCGCATCGCAATC
**Sirt1**	Mouse	GCTGACGACTTCGACGACG	TCGGTCAACAGGAGGTTGTCT
**collagen II**	Mouse	GGGAATGTCCTCTGCGATGAC	GAAGGGGATCTCGGGGTTG
**collagen X**	Mouse	TTCTGCTGCTAATGTTCTTGACC	GGGATGAAGTATTGTGTCTTGGG
**Aggrecan**	Mouse	CCTGCTACTTCATCGACCCC	AGATGCTGTTGACTCGAACCT
